# Genomic characterization of *Citrobacter freundii* strains coproducing OXA-48 and VIM-1 carbapenemase enzymes isolated in leukemic patient in Spain

**DOI:** 10.1186/s13756-019-0630-3

**Published:** 2019-10-29

**Authors:** Rym Lalaoui, Ana Djukovic, Sofiane Bakour, Linda Hadjadj, Jaime Sanz, Miguel Salavert, Jose Luis López-Hontangas, Miguel A. Sanz, Carles Ubeda, Jean-Marc Rolain

**Affiliations:** 1 Aix Marseille Univ, IRD, APHM, MEPHI, Marseille, France; 2 MEPHI, IHU Méditerranée-Infection, 19-21 Boulevard Jean Moulin, 13385 Marseille Cedex 05, France; 3 Centro Superior de Investigación en Salud Pública – FISABIO, Valencia, Spain; 4 Department of Medicine, Hospital Universitari I Politecnic La Fe, University of Valencia, and Centro de Investigación Biomédica en Red de Cáncer, Instituto Carlos III, Valencia, Spain; 5 Hospital La Fe, Valencia, Spain; 6 Centers of Biomedical Research Network (CIBER) in Epidemiology and Public Health, Madrid, Spain

**Keywords:** *Citrobacter freundii*, OXA-48, VIM-1, Carbapenemase, Whole genome sequencing

## Abstract

**Background:**

The emergence of carbapenemase-producing (CP) *Citrobacter freundii* poses a significant threat to public health, especially in high-risk populations. In this study, whole genome sequencing was used to characterize the carbapenem resistance mechanism of three *C. freundii* clinical isolates recovered from fecal samples of patients with acute leukemia (AL) from Spain.

**Materials and methods:**

Twelve fecal samples, collected between 2013 and 2015 from 9 patients with AL, were screened for the presence of CP strains by selecting them on MacConkey agar supplemented with ertapenem (0.5 mg/L). Bacteria were identified by MALDI-TOF mass spectrometry and were phenotypically characterized. Whole genome sequencing of *C. freundii* isolates was performed using the MinION and MiSeq Illumina sequencers. Bioinformatic analysis was performed in order to identify the molecular support of carbapenem resistance and to study the genetic environment of carbapenem resistance encoding genes.

**Results:**

Three carbapenem-resistant *C. freundii* strains (imipenem MIC≥32 mg/L) corresponding to three different AL patients were isolated. Positive modified Carba NP test results suggested carbapenemase production. The genomes of each *C. freundii* tested were assembled into a single chromosomal contig and plasmids contig. In all the strains, the carbapenem resistance was due to the coproduction of OXA-48 and VIM-1 enzymes encoded by genes located on chromosome and on an IncHI2 plasmid, respectively. According to the MLST and the SNPs analysis, all strains belonged to the same clone ST169.

**Conclusion:**

We report in our study, the intestinal carrying of *C. freundii* clone ST169 coproducing OXA-48 and VIM-1 identified in leukemic patients.

## Introduction

In patients with acute leukemia (AL), long duration and repetitive chemotherapy as well as antimicrobial therapy is believed to contribute to occurrence of infections due to multi-drug resistant (MDR) bacteria in this high-risk group [1, 2]. In patients with leukemia, because of therapy of their diseases, bacterial infection with MDR Gram-negative bacteria is a real problem that could be associated with a high rate of mortality and morbidity [2–4].

*Citrobacter freundii* is a gram-negative bacterium which is rarely the causative agent of infections but it has been associated with different infections including respiratory, urinary, gastrointestinal and bloodstream infections [5, 6]. The emergence of MDR *C. freundii,* especially those carbapenemase producing strains, poses a significant threat to public health worldwide, especially in immunocompromised patients such as leukemia patients, which are mostly dependent on antibiotics [5, 6].

Since the development of new generation sequencing technologies, the access to the full genetic bacterial repertoire has become easier and allow a better understanding the emergence of antibiotic resistance genes on a global scale [7, 8].

In this study, we applied the whole-genome sequencing to characterize the antibiotic resistance mechanisms of three carbapenem-resistant *C. freundii* clinical isolates recovered from fecal samples of patients with acute leukemia from Spain.

## Materials and methods

### Study design, bacterial isolates and antimicrobial susceptibility test

The Study design was described in our previous publication [9]. A subset of twelve fecal samples, collected between 2013 and 2015 from 9 patients with AL, were screened for the presence of carbapenemase producing (CP) strains by selecting them on MacConkey agar supplemented with ertapenem (0.5 mg/L) [10]. Cultivated bacteria were identified by matrix-assisted laser desorption ionization–time of flight mass spectrometry (MALDI-TOF MS) (Microflex, Bruker Daltonics, Bremen, Germany). The resistance phenotype of the isolates was evaluated by testing their susceptibly against sixteen antibiotics on Mueller Hinton agar using disk diffusion methodology according to the European Committee on Antimicrobial Susceptibility testing (EUCAST) guidelines (http://www.eucast.org). The minimum inhibitory concentration (MIC) of imipenem was determined using Etest method (AB Biodisk, Sweden), the results were interpreted according to the EUCAST breakpoint. The modified Carba NP test method was used to determine a possible carbapenemase production [11].

### Genetic and genomic characterization

Real-time and standard PCR were performed to screen for the presence of carbapenem resistance genes, including *bla*_OXA-48_, *bla*_KPC_, *bla*_NDM_ and *bla*_VIM_ [12]. Whole genome sequencing of the CP strains was performed using the MinION (Oxford Nanopore Technologies Inc., UK) and the MiSeq (Illumina Inc., San Diego, CA, USA) technologies in order to determine the carbapenemase genes variants, the genetic environment, and the genetic support of these genes. The long-read sequencing data generated by Nanopore and short-read data produced by Illumina sequencing were assembled using SPAdes genome assembler [13]. ARG-ANNOT database available on the ABRicate pipeline and Prokka software were used to identify the antibiotic resistances genes and to annotate genomes, respectively [14, 15]. Genetic environment has been reconstituted by comparing the sequence of genes surrounding the carbapenemase gene against the NCBI database, using blastX parameter.

### Clonal relationship

SNPs analysis (available at https://cge.cbs.dtu.dk/services/CSIPhylogeny/) was conducted to study the genomic difference between the three strains and to determine the possible clonal relationship. In order to determine the sequence type (ST) of isolated strains, Multi Locus Sequence Typing (MLST) analysis was performed in silico using the MLST database (available at https://cge.cbs.dtu.dk/services/MLST/).

### Conjugation experiments and plasmid analysis

Conjugation was conducted on the three *C. freundii* isolates using azide-resistant *Escherichia coli* J53 as a recipient, as previously described [16]. The transconjugants were selected on Luria Bertani (LB) agar (Beckton Dickinson, Le Pont de Claix, France) supplemented with sodium azide (120 μg/ml) and Ertapenem (2 μg/ml). Plasmids analysis was performed in silico. Plasmid incompatibility type was identified using PlasmidFinder database (available at https://cge.cbs.dtu.dk/services/PlasmidFinder/) and Jspecies Web Server was used to calculate the extent of identity between the plasmids [17].

#### Nucleotide sequence accession number

The shotgun whole genome sequence of the three *C. freundii* strains and complete sequence of plasmids have been deposited in NCBI GenBank (GenBank accession number CP038653, CP038654, CP038655, CP038656, CP038657, CP038658, CP038659 and CP038660).

## Results

### Bacterial strains and microbiological tests

Three *C. freundii* strains *(C. freundii*_154, *C. freundii*_565 and *C. freundii*_680) were isolated on selective media from fecal samples of three different leukemic patients (Patient-1, Patient-2 and Patient-3) aged 49, 40, 51, respectively (Table 1). All patients received ciprofloxacin prior sampling and only one (Patient-2) received also meropenem (Table 1). Before samples collection, Patient-1 and Patient-2 received an allogenic transplantation, whereas Patient-3 received a high-intensity chemotherapy. The three strains were resistant to most antibiotics tested (Table 2), including carbapenems with imipenem MIC ≥32 μg/ml. All the strains remained susceptible to doxycycline, colistin, fosfomycin and nitrofurantoin. Positive modified Carba NP test results suggested carbapenemase production.

**Table 1:** Clinical information about leukemic patients harboring cabapenemase-producing *C. freundii*

Patients	Age (years)	Hematological malignancy type	Sample No.	Sampling date	Antimicrobial therapy	Other conditions before sampling	CP bacteria
Patient_1	49	Acute leukemia	154	13/02/2014	Ciprofloxacin	Transplant	CF_154
Patient_2	40	Acute leukemia	565	24/09/2014	Ciprofloxacin, Meropenem	Transplant	CF_565
Patient_3	51	Acute leukemia	680	26/11/2014	Ciprofloxacin	Chemotherapy	CF_680

*CF C. freundii*, *CP* carbapenemase producing

**Table 2:** Analysis of the three *C. freundii* strains isolated from fecal samples of leukemic patients

Strain	Genome size (bp)	GC%	ST	MIC IPM (mg/L)	Sensitive phenotype	Resistance phenotype	Genome composition/ size (bp)	ARGs	Plasmid type	Accession number
CF_154	5,444,819	51,5	169	≥32	DOX, CST, FOF, NIT	AMX, AMC, TZP, CEF, FEP, CRO, ERT, IPM, CIP, AMK, GEN, SXT.	CF154_Chromosome/ 5,143,118	*bla*_CMY-81_, *bla*_OXA-48_, *bla*_TEM-150_, *aac3-IId, aph3-Ia, strA, strB, qnrB38, sulII.*	/	CP038653
							Plasmid_1 (p154_1)/ 296,117	*bla*_CTX-M-9_, *bla*_SHV-12_, *bla*_VIM-1_, *aac6-Ib-cr, aadA1-pm, aadA2, aadB, qnr-A1, catA1, sulI, dfr16.*	IncHI2	CP038654
							Plasmid_2 (p154_2)/ 5584	/	ColRNAI_1	CP038655
CF_565	5,471,065	51,5	169	≥32	DOX, CST, FOF, NIT	AMX, AMC, TZP, CEF, FEP, CRO, ERT, IPM, CIP, AMK, GEN, SXT.	CF565_Chromosome/ 5,207,876	*bla*_CMY-81_, *bla*_OXA-48_, *bla*_TEM-150_, *aac3-IId, aph3-Ia, strA, strB, qnrB38, sulII.*	/	CP038656
							Plasmid_1 (p565_1)/ 263,189	*bla*_CTX-M-9_, *bla*_SHV-12_, *bla*_VIM-1_, *aac6-Ib-cr, aadA1-pm, aadA2, qnr-A1, catA1, sulI, dfr16.*	IncHI2	CP038657
CF_680	5,557,664	51,4	169	≥32	DOX, CST, FOF, NIT	AMX, AMC, TZP, CEF, FEP, CRO, ERT, IPM, CIP, AMK, GEN, SXT.	CF680_Chromosome/ 5,167,642	*bla*_CMY-81_, *bla*_OXA-48_, *bla*_TEM-150_, *aac3-IId, aph3-Ia, strA, strB, qnrB38, sulII.*	/	CP038658
							Plasmid_1 (p680_1)/ 385,971	*bla*_CTX-M-9_, *bla*_OXA-9_, *bla*_SHV-12_, *bla*_TEM-150_, *bla*_VIM-1_, *aac6-Ib-cr, aadA1-pm, aadA2, aadB, qnr-A1, catA1, sulI, dfr16.*	IncHI2	CP038659
							Plasmid_2 (p680_2)/ 4051	/	ColRNAI_1	CP038660

*AMX* Amoxicillin, *AMC* Amoxicillin/clavulanic acid, *TZP* Piperacillin + Tazobactam, *CEF* Cephalothin, *FEP* Cefepime, *CRO* Ceftriaxone, *ERT* Ertapenem, *IPM* Imipenem, *CIP* Ciprofloxacin, *AMK* Amikacin, *GEN* Gentamicin, *DOX* Doxycycline, *CST*; Colistin, *FOF* Fosfomycin, *NIT* Nitrofurantoin, *SXT* Sulfamethoxazole/trimethoprim, *MIC* Minimum Inhibitory Concentration, *ARGs* Antibiotic resistance genes, *ST* Sequence Type

### Genetic, genomic and molecular epidemiology analysis

**Fig. 1:**
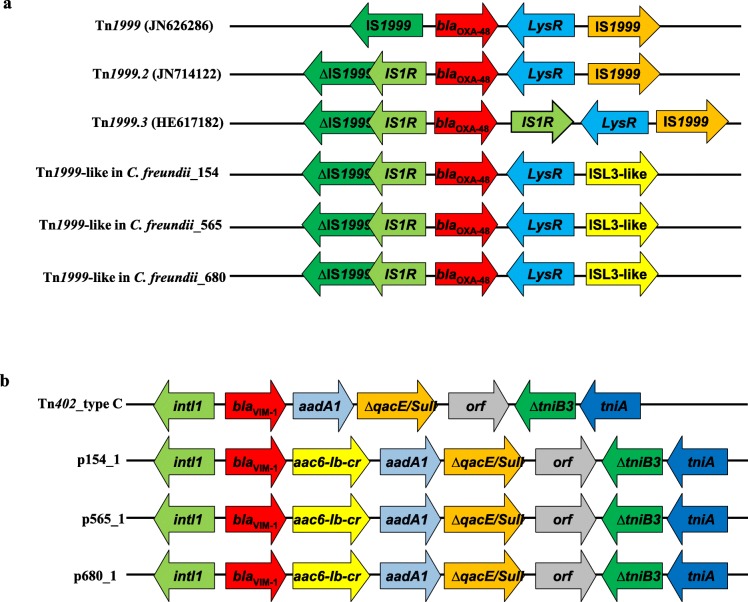
**a** Schematic representation of the genetic environment of the *bla*_OXA-48_ gene located on chromosome and its comparison with the Tn*1999* transposon (JN626286) and its variant Tn*1999.2* (JN714122) and Tn*1999.3* (HE617182). **b** Schematic representation of genes surrounded the *bla*_VIM-1_ gene and the comparison of this genetic environment with that one identified in the Tn*402*-type C

The genome size of CP *C. freundii* strains obtained after assembly ranged from 5′443’022 and 5′471’065 bp (including chromosome and plasmids for each strains) (Table 2). According to PCR results and genome analysis, carbapenem resistance in these strains was due to the co-production of OXA-48 and VIM-1 carbapenemase enzymes. Resistome analysis showed the presence of genes encoding for resistance to β-lactams, aminoglycosides, quinolones, sulfonamides, trimethoprim and chloramphenicol antibiotics families (Table 2). The gene encoding OXA-48 enzyme was located on the chromosome in the three strains tested and surrounded by the almost similar structures that compose the Tn*1999*.2 transposon (ΔTn1999/IS*1R*-*bla*_OXA-48_-*LysR*-*orf*-ISL3-like) (Fig. 1.a). Unlike the *bla*_OXA-48_ gene, *bla*_VIM-1_ gene was located in a IncHI-2 plasmid in all the strains. This gene was located in a class 1 integron that contains a new cassette array (*intI1*–*bla*_*VIM-1*_-*aac6-Ib-cr*-*aadA1*-*qqcEΔ1*/*sul1-ΔtniB3-tniA)* (Fig. 1b). According to the MLST analysis, all strains belonged to the same sequence type, ST169. The SNPs analysis found between 10 and 19 SNPs on average between the three isolates suggesting that these ST169 strains belonged to the same clone.

### Plasmid conjugation

Conjugation experiments failed for the three strains tested, whereas, in silico plasmids analysis showed the presence of different protein implicated in conjugal transfer and pilus formation but the absences of the plasmid transfer origin (oriT). The comparison of the average nucleotide identity between the three plasmids harboring *bla*_VIM-1_ gene showed that plasmid p154_1 shared 99.99% of similarity with the plasmid p565_1, whereas p680_1 shared 99.94% of similarity with the two other plasmids.

## Discussion

Carbapenemase production in *C. freundii* is poorly documented, only a few studies reported the expression of such enzymes in this species [6, 18–20]. The coproduction of carbapenemase enzymes was already described in some *Enterobacteriaceae* species such as *Klebsiella pneumoniae* (KPC-2 + VIM-2 or NDM-1 + VIM-1) [19, 21, 22] and *Enterobacter cloacae* (NDM-1 + VIM-1) [19] as well as in *C. freundii* (KPC-2 + NDM-1, NDM-1 + VIM-1) [19, 20]. In our study, this situation was observed in three carbapenem resistant *C. freundii* strains detected in fecal samples of three AL patients where, interestingly, two of them didn’t received carbapenem as antimicrobial therapy. This suggest that the carbapenem resistance in this context may not be due to a selection pressure with this antibiotic family but it could have been selected by the use of other antibiotic families or by the presence of a carbapenem-resistant clone in the hematological ward, which would explain this current situation.

In Spain, OXA-48 and VIM-1 enzymes are the most prevalent carbapenemase enzymes reported especially in *E. coli*, *E. cloacae* and *K. pneumoniae* [18]. The coproduction of these two carbapenemase enzymes by *C. freundii* species was reported in only three studies over the world, and only one reported this detection in hematological malignancies patients [18, 19, 23]. During an unrestricted and non-mandatory national Spanish Antibiotic Resistance Surveillance Programme, undertaken between 2013 and 2015, it has been noted a progressive increase in the rate of *Citrobacter* spp. Carbapenemase-producers, including *C. freundii* species, in Spanish hospital from 1.3% in 2013 to 1.5% in 2015 [18].

The gene encoding OXA-48 enzyme was mainly related to the Tn*1999* transposon and to its variants [24]. Our study showed that the *bla*_OXA-48_ gene was located on the chromosome in all strains tested and that its genetic environment was almost identical to that described in Tn*1999.2* variant (Table 2, Fig. 1a) [24]. Indeed, the only difference resides downstream of the *bla*_OXA-48_ gene, where this gene was flanked by an ISL3-like in our three *C. freundii* instead of IS*1999* described in Tn*1999.2* variant (Fig. 1a).

*bla*_VIM-1_ gene was widely detected in different class 1 integrons such as In110 or In113 [25]. In the study conducted by Arana et al in Spain, *bla*_VIM-1_ identified in their *C. freundii* strains was located on class 1 integrons which include other antibiotics resistant genes such as *aacA4, dfrB1, aadA1*, and *catB2*genes [18]. Our study also reports the localization of the *bla*_VIM-1_ gene in a class 1 integrons, which contains a new gene cassette, composed of the *bla*_VIM-1_, *aac6-Ib-cr* (conferring resistance to both aminoglycosides and quinolones), *aadA1* (conferring resistance to aminoglycosides) as well as the classic *sulI* gene (Fig. 1b). The structure of the integrons class 1 type identified in our strains looks like the defective Tn*402* transposon (type C) carrying the *tni* module, ∆*tniB* and *tniA*, reported in the literature (Fig. 1b) [26].

In our study, the plasmid carrying the *bla*_VIM-1_ gene identified cannot conjugate, thus excluding the possibility of plasmid dissemination between patients. Moreover, MLST and SNPs analysis showed that the three *C. freundii* strains belonged to the same ST169 clone, which leads us to hypothesize a possible clonal spread of carbapenem-resistant strains in the hematology department.

In this present study, the three *C. freundii* coproducing OXA-48 and VIM-1 carbapenemase enzymes were isolated in a context of digestive carrying and not infectious. It has been shown that in hematological patients, colonization of the digestive tract by carbapenem-resistant bacteria constitutes a risk in the development of infections with these bacteria [27–29]. Despite the fact that our isolates exhibited a high resistance profile, some antibiotics remained active on these bacteria such as doxycycline, colistin, fosfomycin or nitrofurantoin.

## Conclusion

This study reports the clonal spread of *C. freundii* ST169 exhibiting a rare phenotype of co-production of two carbapenemases, namely OXA-48 and VIM-1 enzymes, detected in the digestive tract of patients with acute leukemia. In our opinion, a systematic screening of digestive carriage of antibiotics resistant bacteria would be a great solution to prevent the occurrence of infections due to such bacteria and to control the spread of antibiotic resistance genes, especially within high risk populations.

## Data Availability

Not applicable.

## Abbreviations

AL: Acute leukemia
ARG-ANNOT: Antibiotic resistance gene-annotation
CP: Carbapenemase producing
EUCAST: European Committee on Antimicrobial Susceptibility Testing
LB: Lucia Bertani
MALDI-TOF MS: Matrix-assisted laser desorption and ionization time-of-flight mass spectrometry
MDR: Multi drug resistant
MIC: Minimum inhibitory concentration
MLST: Multi-locus sequence typing
NCBI: National Center for Biotechnology Information
PCR: Polymerase chain reaction
SNPs: Single nucleotide polymorphisms
ST: Sequence type

## References

[1] Alp S, Akova M. Antibacterial resistance in patients with hematopoietic stem cell transplantation. Mediterr J Hematol Infect Dis. 2017;9:e2017002. 10.4084/MJHID.2017.002 PMC5224809 28101308

[2] Trubiano JA, Worth LJ, Thursky KA, Slavin MA (2015). The prevention and management of infections due to multidrug resistant organisms in haematology patients. Br J Clin Pharmacol.

[3] Baker TM, Satlin MJ (2017). The growing threat of multidrug-resistant gram-negative infections in patients with hematologic malignancies. Leuk Lymphoma.

[4] Blennow Ola, Ljungman Per (2015). The challenge of antibiotic resistance in haematology patients. British Journal of Haematology.

[5] Yang L, Peihan L, Beibei L, Xiaofeng H, Jinhui L, Jing X (2018). Multidrug-resistant Citrobacter freundii ST139 co-producing NDM-1 and CMY-152 from China. Sci Rep.

[6] Ouyang J, Xiong Z, Yang B, Liu Z, Sun F, Zhou D (2018). Comparative genomics of five different resistance plasmids coexisting in a clinical multi-drug resistant Citrobacter freundii isolate. Infect Drug Resist.

[7] Bakour Sofiane, Sankar Senthil Alias, Rathored Jaishriram, Biagini Philippe, Raoult Didier, Fournier Pierre-Edouard (2016). Identification of virulence factors and antibiotic resistance markers using bacterial genomics. Future Microbiology.

[8] Hadjadj L, Baron SA, Diene SM, Rolain J-M (2019). How to discover new antibiotic resistance genes?. Expert Rev Mol Diagn.

[9] Lalaoui Rym, Djukovic Ana, Bakour Sofiane, Sanz Jaime, Gonzalez-Barbera Eva M., Salavert Miguel, López-Hontangas Jose Luis, Sanz Miguel A., Xavier Karina B., Kuster Bernhard, Debrauwer Laurent, Ubeda Carles, Rolain Jean-Marc (2019). Detection of plasmid-mediated colistin resistance, mcr-1 gene, in Escherichia coli isolated from high-risk patients with acute leukemia in Spain. Journal of Infection and Chemotherapy.

[10] Bachiri T, Bakour S, Lalaoui R, Belkebla N, Allouache M, Rolain JM (2017). Occurrence of carbapenemase-producing enterobacteriaceae isolates in the wildlife: first report of OXA-48 in wild boars in Algeria. Microb Drug Resist.

[11] Bakour S, Garcia V, Loucif L, Brunel J-M, Gharout-Sait A, Touati A (2015). Rapid identification of carbapenemase-producing Enterobacteriaceae, Pseudomonas aeruginosa and Acinetobacter baumannii using a modified Carba NP test. New Microbes New Infect.

[12] Mellouk FZ, Bakour S, Meradji S, Al-Bayssari C, Bentakouk MC, Zouyed F (2017). First Detection of VIM-4-Producing Pseudomonas aeruginosa and OXA-48-Producing Klebsiella pneumoniae in Northeastern (Annaba, Skikda) Algeria. Microb Drug Resist.

[13] Bankevich A, Nurk S, Antipov D, Gurevich AA, Dvorkin M, Kulikov AS (2012). SPAdes: A New Genome Assembly Algorithm and Its Applications to Single-Cell Sequencing. J Comput Biol.

[14] Gupta SK, Padmanabhan BR, Diene SM, Lopez-Rojas R, Kempf M, Landraud L (2014). ARG-ANNOT, a New Bioinformatic Tool To Discover Antibiotic Resistance Genes in Bacterial Genomes. Antimicrob Agents Chemother.

[15] Seemann T (2014). Prokka: rapid prokaryotic genome annotation. Bioinformatics.

[16] Lalaoui R, Bakour S, Livnat K, Assous M, Diene S, Rolain J (2019). Spread of Carbapenem and Colistin-resistant Klebsiella pneumoniae ST512 clinical isolates in Israel: a cause for vigilance. Microb Drug Resist.

[17] Richter M, Rosselló-Móra R, Oliver Glöckner F, Peplies J (2016). JSpeciesWS: a web server for prokaryotic species circumscription based on pairwise genome comparison. Bioinformatics.

[18] Arana DM, Ortega A, González-Barberá E, Lara N, Bautista V, Gómez-Ruíz D (2017). Carbapenem-resistant Citrobacter spp. isolated in Spain from 2013 to 2015 produced a variety of carbapenemases including VIM-1, OXA-48, KPC-2, NDM-1 and VIM-2. J Antimicrob Chemother.

[19] Bedenić B, Sardelić S, Luxner J, Bošnjak Z, Varda-Brkić D, Lukić-Grlić A (2016). Molecular characterization of class b carbapenemases in advanced stage of dissemination and emergence of class d carbapenemases in Enterobacteriaceae from Croatia. Infect Genet Evol.

[20] Feng J, Qiu Y, Yin Z, Chen W, Yang H, Yang W (2015). Coexistence of a novel KPC-2-encoding MDR plasmid and an NDM-1-encoding pNDM-HN380-like plasmid in a clinical isolate of Citrobacter freundii. J Antimicrob Chemother.

[21] Papagiannitsis CC, Malli E, Florou Z, Sarrou S, Hrabak J, Mantzarlis K (2017). Emergence of sequence type 11 *Klebsiella pneumoniae* coproducing NDM-1 and VIM-1 metallo-β-lactamases in a Greek hospital. Diagn Microbiol Infect Dis.

[22] Falco A, Ramos Y, Franco E, Guzmán A, Takiff H (2016). A cluster of KPC-2 and VIM-2-producing Klebsiella pneumoniae ST833 isolates from the pediatric service of a Venezuelan Hospital. BMC Infect Dis.

[23] Jayol A, Poirel L, Dortet L, Nordmann P (2016). National survey of colistin resistance among carbapenemase-producing Enterobacteriaceae and outbreak caused by colistin-resistant OXA-48-producing *Klebsiella pneumoniae*, France, 2014. Euro Surveill.

[24] Machuca J, López-Cerero L, Fernández-Cuenca F, Mora-Navas L, Mediavilla-Gradolph C, López-Rodríguez I, et al. OXA-48-like-producing Klebsiella pneumoniae in southern Spain in 2014–2015. Antimicrob Agents Chemother. 2019;63:e01396-18. 10.1128/AAC.01396-18 PMC6325174 30323046

[25] Porres-Osante N, Azcona-Gutiérrez JM, Rojo-Bezares B, Undabeitia E, Torres C, Sáenz Y (2014). Emergence of a multiresistant KPC-3 and VIM-1 carbapenemase-producing Escherichia coli strain in Spain. J Antimicrob Chemother.

[26] Tato M, Coque TM, Baquero F, Cantón R (2010). Dispersal of carbapenemase blaVIM-1 gene associated with different Tn402 variants, mercury transposons, and conjugative plasmids in Enterobacteriaceae and Pseudomonas aeruginosa. Antimicrob Agents Chemother.

[27] Jaiswal SR, Gupta S, Kumar RS, Sherawat A, Rajoreya A, Dash SK (2018). Gut colonization with carbapenem-resistant enterobacteriaceae adversely impacts the outcome in patients with hematological malignancies: results of a prospective surveillance study. Mediterr J Hematol Infect Dis.

[28] Fanci R, Sica S, Cattaneo C, Fianchi L, Busca A, Trecarichi EM (2016). Bloodstream infections caused by Klebsiella pneumoniae in onco-hematological patients: clinical impact of carbapenem resistance in a multicentre prospective survey. Am J Hematol.

[29] Andria N, Henig O, Kotler O, Domchenko A, Oren I, Zuckerman T (2015). Mortality burden related to infection with carbapenem-resistant gram-negative bacteria among haematological cancer patients: a retrospective cohort study. J Antimicrob Chemother.

